# Pre-flight body weight effects on urinary calcium excretion in space

**DOI:** 10.1038/s41526-023-00291-2

**Published:** 2023-06-14

**Authors:** Semran Thamer, Mirjana Stevanovic, Jay C. Buckey

**Affiliations:** 1 Geisel School of Medicine at Dartmouth, 1 Rope Ferry Rd, Hanover, NH 03755 USA; 2 Space Medicine Innovations Laboratory, Geisel School of Medicine at Dartmouth, One Medical Center Drive, Lebanon, NH 03756 USA

**Keywords:** Risk factors, Physiology

## Abstract

Microgravity-induced bone loss increases urinary calcium excretion which increases kidney stone formation risk. Not all individuals show the same degree of increase in urinary calcium and some pre-flight characteristics may help identify individuals who may benefit from in-flight monitoring. In weightlessness the bone is unloaded, and the effect of this unloading may be greater for those who weigh more. We studied whether pre-flight body weight was associated with increased in-flight urinary calcium excretion using data from Skylab and the International Space Station (ISS). The study was reviewed and approved by the National Aeronautics and Space Administration (NASA) electronic Institutional Review Board (eIRB) and data were sourced from the Longitudinal Study of Astronaut Health (LSAH) database. The combined Skylab and ISS data included 45 participants (9 Skylab, 36 ISS). Both weight and day in flight were positively related to urinary calcium excretion. There was also an interaction between weight and day in flight with higher weight associated with higher calcium excretion earlier in the mission. This study shows that pre-flight weight is also a factor and could be included in the risk assessments for bone loss and kidney stone formation in space.

## Introduction

Prolonged microgravity exposure during long-duration human space flight results in a physiological imbalance of bone remodeling^1^. Weightlessness unloads the skeleton so that bone resorption is favored over bone formation^1^. This results in significant loss of bone mineral density, at a rate of approximately 1–1.5% per month in weight-bearing areas like the hip and lumber spine, which is only partially responsive to non-pharmacological countermeasures^2,3^. Additionally, microgravity-induced bone resorption leads to calcium loss from bone which enters the systemic circulation and is excreted in the urine^4^. As a result, the increased urinary calcium can supersaturate leading to an increased risk of kidney stone formation.

Microgravity-induced bone loss and kidney stone risk are currently unresolved health risks for long-duration space travelers^5^. Generally, crew members engage in physical exercise for fifteen hours a week to reduce these risks^6^. On long-duration International Space Station (ISS) flights, these risks are currently being controlled by careful astronaut selection, attention to hydration, as well as an exercise program^7,8^. The risk of bone loss and kidney stone formation has not been completely eliminated through these measures^9^.

On Earth, a variety of factors influence these risks including sex, dietary intake, race, genetic predisposition, lifestyle, and comorbidities^10,11^. There is also inherent variability in the amount of urinary calcium healthy as well as diseased individuals normally excrete^12,13^. The normal range for urinary calcium is wide, and its limits are not well described^14^. This suggests that not all individuals are at equal risk of losing bone or forming kidney stones in space. Identifying those at highest risk of these complications may help inform measures to reduce these risks. As commercial and lunar spaceflight is projected to become increasingly popular, the risk of bone loss and kidney stone formation may increase due to less stringent screening and smaller spacecraft with limited countermeasure capability. In contrast to rigorous astronaut selection among career-astronauts, medical screening for commercial spaceflight passengers may be less aggressive^15^. As a result, passengers among the general population who have risk factors for bone loss or kidney stones may be permitted to fly in space. This could pose significant ethical and health implications if these risks are not mitigated among passengers on long-duration spaceflights.

Physical exercise, an established countermeasure to reduce—albeit not completely—the risk of bone loss and kidney stone formation, may not be feasible for lunar and capsule flights where space and resources are limited^16^. While several pharmacologic as well as other non-pharmacological countermeasures have been proposed, one of the biggest challenges may be identifying those at highest risk, perhaps by monitoring urinary calcium excretion. When bone loss occurs, urinary calcium excretion increases which can be measured and used to assess an individual’s level of bone resorption and risk of kidney stone formation in real time^17,18^. This can be used to guide frequency of non-pharmacological countermeasures or dosing of pharmacological measures like bisphosphonates or potassium citrate—both of which have dose-dependent effects and are currently being studied as potential countermeasures^19,20^. Therefore, by using urinary calcium excretion to identify high-risk populations, targeted countermeasures can be individualized in real-time.

Previous research on Earth has demonstrated that weight plays a role in influencing urinary calcium excretion, kidney stone formation, and bone mineral density^21–23^. While the mechanism has not yet been fully elucidated, greater body weight, waist circumference, and body mass index have all been associated with increased urinary calcium excretion, bone loss, and kidney stone formation risk in terrestrial studies^10,22,24^. However, there is a scarcity of literature identifying the relationship of body weight and urinary calcium excretion in space. Therefore, in this research, we examined data from the Skylab and ISS programs to assess the relationship between pre-flight weight and in-flight urinary calcium excretion.

## Methods

### Study design

Data from the Skylab and ISS programs were obtained from the Longitudinal Study of Astronaut Health (LSAH) database. All ISS data were de-identified, while the Skylab data had previously been published in identifiable form. This study was reviewed and approved by the National Aeronautics and Space Administration (NASA) and the Johnson Space Center (JSC) electronic Institutional Review Board (eIRB). Longitudinal in-flight 24 h urinary calcium excretion measurements (mg/day) were available for 9 and 36 individuals from the Skylab and ISS programs, respectively. For both programs, information on pre-flight weight (kg) was available. Pre-flight urinary calcium excretion measurements were only available for the Skylab program.

### Statistical analyses

To test the effect of baseline urinary calcium excretion on in-flight urinary calcium excretion, we fit a linear mixed effects model using Skylab data only. Weight and day of flight were included as covariates, and subject identification (ID) was modeled as a random effect. We next examined the effect of pre-flight weight, day of flight, and their interaction on in-flight urinary calcium excretion using the combined Skylab and ISS data. We fit a linear mixed effects model that included program (Skylab/ISS) as a covariate and subject ID as a random effect. In both models, the random effect included both intercepts and slopes. All statistical analyses were performed in R. All statistical tests were two-sided and statistical significance was considered at *p* < 0.05.

### Reporting summary

Further information on research design is available in the Nature Research Reporting Summary linked to this article.

## Results

### Data analyses

**Fig. 1:**
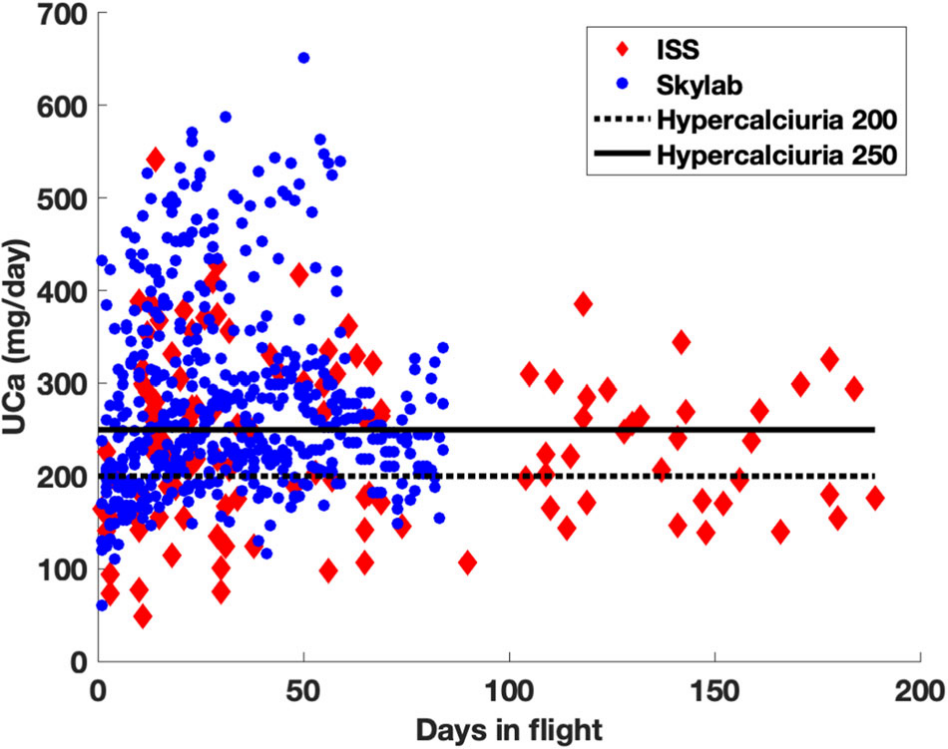
Plot of the combined dataset. Circles represent Skylab, triangles represent ISS. Blue symbols were less than the median weight and red symbols were above.

**Fig. 2:**
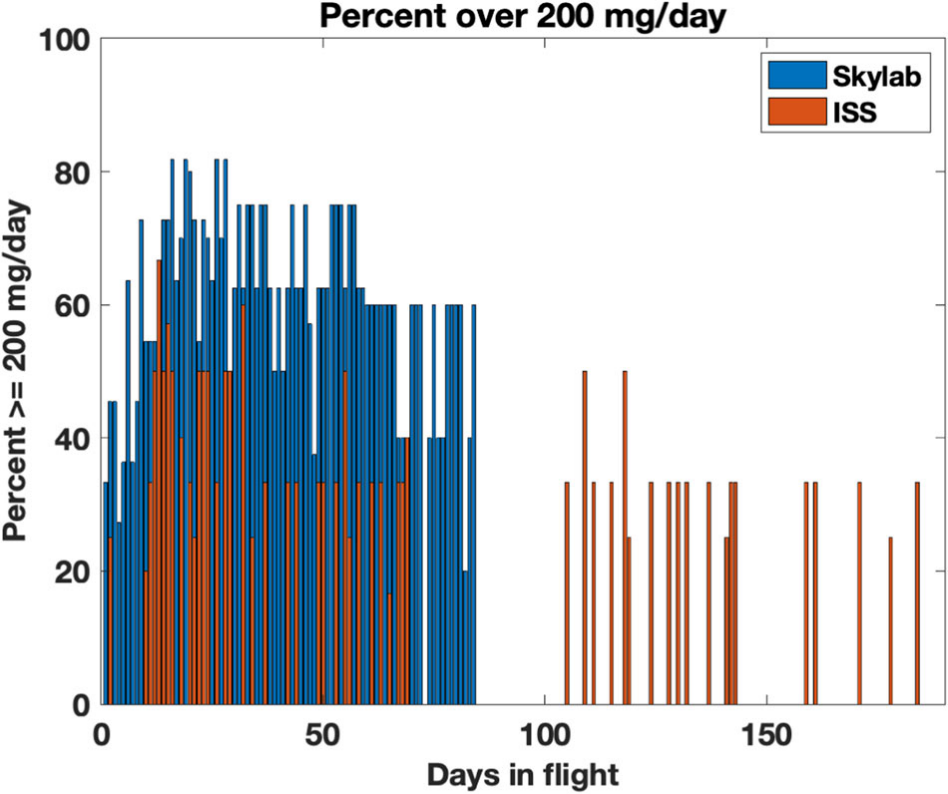
Plot of urinary calcium excretion in relation to days in flight. Percent of urinary calcium excretion at 200 mg per day or greater.

**Fig. 3:**
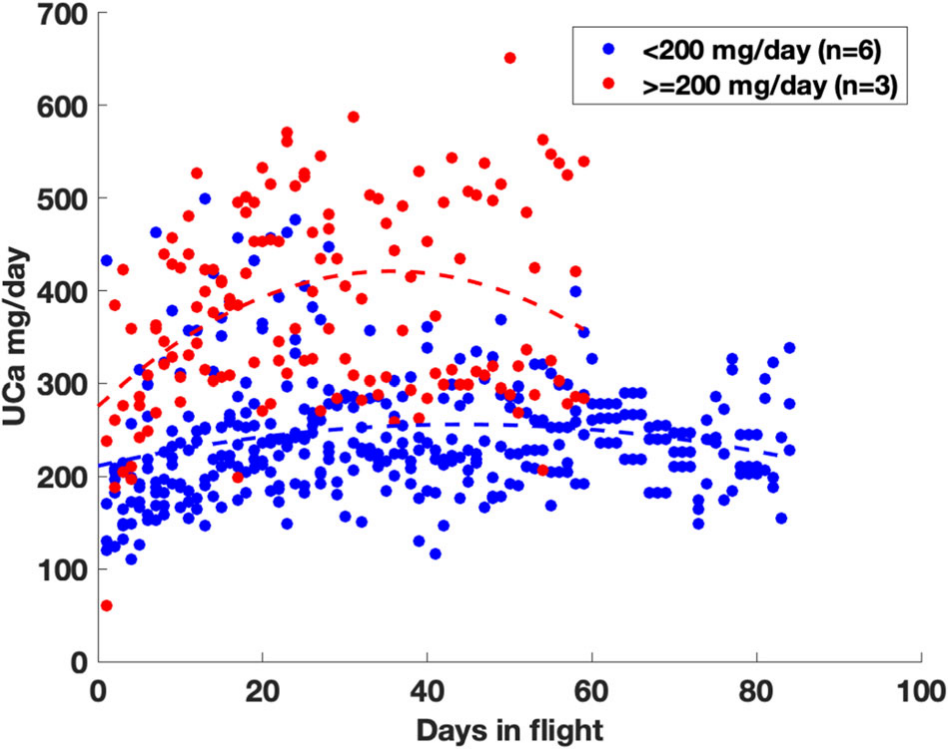
Baseline pre-flight urinary calcium excretion versus in-flight urinary calcium excretion among Skylab participants. Baseline data for ISS participants were not available. Red and blue circles represent subjects with high and low baseline urinary calcium excretion, respectively. The dashed lines represent lines of best fit (second order polynomials) for those two sets of subjects.

The mean in-flight urinary calcium excretion was 273 mg/day (SD = 100) for all 628 collections. The mean urinary calcium excretion was 281 mg/day (SD = 101) using Skylab data and 238 mg/day (SD = 90) for ISS data (Figs. 1, 2). Table 1 shows the characteristics of the dataset. Based on the model using Skylab data only, there was a statistically significant association between baseline urinary calcium excretion and urinary calcium excretion during flight controlling for pre-flight weight and day in flight (coefficient = 1.1; 95% CI: 0.47, 1.8; *p* = 0.018) (Fig. 3). In this model the effect of weight was not significant.

**Table 1:** Characteristics of the dataset.

	Skylab	ISS	Overall	*P*-value (Skylab vs. ISS)
*N*	9	36	45	
Mean Urinary calcium mg/day	281 ± 101	238 ± 90	273 ± 100	0.019
Mean Weight (kg)	72.3 ± 8.8	77.1 ± 11.6	76.1 ± 11.2	0.254
Mean observations/crewmember	56 ± 25	3.4 ± 1.4	14 ± 24	< 0.001

Using the combined dataset, we found a statistically significant association between pre-flight weight and urinary calcium excretion during flight (coefficient = 2.8; 95% CI: 0.38, 5.2; *p* = 0.029) (Table 2). The association between day in flight and urinary calcium excretion during flight approached significance (coefficient = 3.8; 95% CI: 0.21, 7.3; *p* = 0.055). The interaction term between pre-flight weight and day in flight also approached statistical significance (coefficient = −0.047, 95% CI: −0.093, −0.00095; *p* = 0.064). While the magnitude of the coefficient was small, the coefficient was negative, which could suggest that as time increases, the slope of the association between pre-flight weight and urinary calcium excretion during flight decreases, indicating that increased weight has a greater effect on urinary calcium excretion in the earlier days in flight.

**Table 2:** Results from linear mixed effect model for the combined Skylab/ISS dataset.

Factor	Coefficient	SE^1^	Tstat	DF^2^	*P*-value
Weight (kg)	2.8	1.2	2.3	40	0.029
Days in flight	3.8	1.8	2.1	15	0.055
Weight*Days in flight	−0.047	0.023	−2	15	0.064
Mission (ISS reference)	26	31	0.85	31	0.4

^1^Standard Error, ^2^Degrees of Freedom.

## Discussion

In this study, greater pre-flight body weight was associated with an increased risk of higher in-flight urinary calcium excretion. Increased body weight also showed a tendency to be associated with a more rapid rise in urinary calcium excretion. These findings suggest that passengers with an increased pre-flight body weight may be at an increased risk of bone loss and kidney stone formation than lower weight counterparts.

To the best of our knowledge, this is the first study that has identified pre-flight weight as a risk factor for increased in-flight urinary calcium. The findings of this study corroborate previous research evaluating factors influencing urinary calcium excretion among healthy terrestrial subjects, including body weight^14,25–27^. Weight loss can lead to bone resorption and decreased bone mineral density^28–30^. Those with higher baseline body weight are especially prone to this phenomenon^31,32^. Additionally, rapid as opposed to gradual weight loss potentiates this effect^28,33^. Weightlessness in space unloads the skeleton at a much more rapid rate and degree than weight loss does on Earth. In turn, it is possible that skeletal unloading from weightlessness may explain the relationship between weight and urinary calcium excretion observed in this study. Those with greater pre-flight weight may therefore be more prone to skeletal unloading and have a greater effect from reducing their skeletal load in weightlessness.

Previous research has shown that urinary calcium excretion can serve as a marker of bone loss and kidney stone risk while in space^34–36^. However, identifying which passengers may benefit from in-flight urinary calcium monitoring has not been well established. Based on the findings of this study, pre-flight medical screening for spaceflight passengers may incorporate body weight to select individuals for whom closer monitoring of urinary calcium levels may be useful while they are in microgravity.

While our findings suggest that higher weight is associated with increased urinary calcium excretion during flight, the effect was not profound. Based on this analysis, a 10 kg increase in body weight would translate into a 28 mg/day increase in urinary calcium excretion, which is an approximate 10% increase based on the average overall urinary calcium excretion. This suggests that other factors may play a more substantial role in influencing calcium excretion. Nevertheless, these results could provide useful information for risk stratification. For the same baseline level of urinary calcium excretion, a heavier individual may be running a higher risk of stone formation. While weight is a significant risk factor for kidney stones, many other factors including genetic predisposition, diet, and hydration, exist^11^. Therefore, weight alone may not reliably predict risk of kidney stones, bone loss, or urinary calcium excretion in space. Instead, weight may be used as one of the many factors that may need to be considered to establish an individual’s risk profile.

This study has some limitations. The amount of data available was limited and the number of individuals studied was small and may not reflect the population of commercial spacefarers. Full data on gender was not available. Further research with larger sample sizes is needed to validate the findings of this study since body weight is not the only factor affecting urinary calcium excretion. Additionally, the association between baseline urinary calcium excretion and urinary calcium excretion during flight was tested using measurements for only 9 individuals from the Skylab program because the ISS data did not contain data for pre-flight urinary calcium excretion. While we detected a statistically significant association between baseline urinary calcium excretion and in-flight urinary calcium excretion, the small sample size limited our statistical power to detect an association between pre-flight weight and in-flight urinary calcium excretion during flight using just the Skylab data. To test this association, we therefore combined Skylab and ISS data.

Combining data from the Skylab and ISS programs increased our sample size to 45 participants. This also created another limitation, namely that the two programs differ. There may be differences in diet and the use of exercise as a countermeasure to prevent bone loss in space, which could affect urinary calcium excretion during flight but are difficult to account for. We attempted to adjust for these differences statistically by controlling for mission in our model using the combined data, but residual confounding remains a possibility.

Finally, 19 out of 45 participants had 3 or fewer measurements, and 11 participants had only 1 or 2 measurements. As a result, individuals who could be considered outliers, for example individuals with relatively low weight but very high levels of baseline urinary calcium excretion levels, could have a fairly large impact on the results of our analysis given that we were not able to control for baseline urinary calcium excretion in the combined analysis. Despite these limitations, this study highlights the utility for using pre-flight weight for assessing the risk for increased urinary calcium excretion in space using actual space flight data. Higher weight individuals could be targeted for urinary calcium measurements in space.

The risk of bone loss and kidney stone formation are significant barriers that must be overcome for the future success of long duration spaceflight. This will be especially critical as the general population, including those with endogenous risk factors for increased urinary calcium excretion, will be traveling in space. Loss of bone mineral density leads to an increased risk of fractures as well as kidney stones—either of these events happening in-flight can be problematic or even detrimental in the resource-confined environment of spaceflight. Therefore, targeting individuals at an increased baseline risk for these issues with close monitoring of urinary calcium excretion can mitigate these adverse events. Small, low power, point-of-care devices are being developed that could make these measurements. Further research evaluating the efficacy of using body weight to reduce these risks is needed to validate the utility of using these factors in stratifying bone loss and kidney stone risk.

## Supplementary information

Reporting Summary

## Data Availability

The data that support the findings of this study are available from the corresponding author, [ST], upon reasonable request.

## Code availability

The code that support the findings of this study are available from the corresponding author, [ST], upon reasonable request.
